# Priming Engineers to Think About Sustainability: Cognitive and Neuro-Cognitive Evidence to Support the Adoption of Green Stormwater Design

**DOI:** 10.3389/fnins.2022.896347

**Published:** 2022-05-11

**Authors:** Mo Hu, Tripp Shealy

**Affiliations:** Department of Civil and Environmental Engineering, Virginia Tech, Blacksburg, VA, United States

**Keywords:** fNIRS, prefrontal cortex, engineering design, sustainability, priming

## Abstract

Green infrastructure is the application of nature-based solutions like bioswales, rain gardens, and permeable pavements to reduce flooding in urban areas. These systems are underutilized in the design of the built environment. A barrier to their implementation is that design engineers tend to discount the tangential benefits of these greener systems and overweigh the associated risks. This study tested whether priming engineers to think about the environmental and social sustainability benefits of green infrastructure can influence what attributes engineers consider and how they weigh these attributes during the design decision-making process. Forty engineering students trained in stormwater design were asked to evaluate the implementation of a conventional stormwater design option and a green stormwater design option. Their preferred design option was recorded and the changes in their neuro-cognition were measured using functional near infrared-spectroscopy. Half of the engineers were asked to first consider the potential outcomes of these options on the environment and the surrounding community. Priming engineers to first consider environmental and social sustainability before considering the cost and risk of each option, significantly increased the perceived benefits the engineers believed green infrastructure could provide. The priming intervention also increased the likelihood that engineers would recommend the green infrastructure option. The engineers primed to think about environmental and social sustainability exhibited significantly lower oxy-hemoglobin in their ventrolateral, dorsolateral, and medial prefrontal cortex through multiple phases of the judgment and decision-making process. The intervention appears to increase cognitive representativeness or salience of the benefits for green infrastructure when engineers evaluate design alternatives. This relatively low-cost intervention, asking engineers to consider environmental and social sustainability for each design alternative, can shift engineering decision-making and change neuro-cognition.

## Introduction

Barriers in human cognition are cited as the most profound inhibitor to the implementation of green infrastructure (Dhakal and Chevalier, 2017; Li et al., 2020). Green infrastructure is a stormwater management strategy that restores and mimics natural water systems. Green infrastructure manages stormwater using engineered green space (Benedict and MacMahon, 2002). Plants and soils used in green infrastructure absorb and filter excessive stormwater runoff through a natural hydrological process (Foster et al., 2011). Green infrastructure systems are different than conventional stormwater systems that rely on holding tanks and barrels to capture and then filter stormwater. Designing and building conventional stormwater systems are expensive and increasingly ineffective due to capacity limits and the connection to complex, overly-burdened combined sewer overflow systems (Dhakal and Chevalier, 2017; Ghofrani et al., 2017; Keeler et al., 2019).

Green infrastructure provides additional benefits to the environment and surrounding communities compared to conventional systems (Krivtsov et al., 2022). For example, green infrastructure contributes to carbon sequestration and climate change mitigation (Adkins et al., 2015). Added green space can also enhance quality of life for communities and increase the value of surrounding properties (Lee and Anderson, 2013). Even with these known benefits, green infrastructure is not implemented frequently enough to create more sustainable stormwater infrastructure systems (Dhakal and Chevalier, 2017; Hu and Shealy, 2020). The cognitive processes decision-makers use in valuing such sustainability benefits and weighing the costs and potential risks limit its implementation (Dhakal and Chevalier, 2017; Hu and Shealy, 2020; Li et al., 2020).

Engineers often lack adequate information about the benefits of green infrastructure. This creates unbalanced priorities that tend to favor more conventional design options (Copeland, 2016; Dhakal and Chevalier, 2017). For example, monetizing the value of green systems is not straightforward (Vesely, 2007; Dhakal and Chevalier, 2017; Keeler et al., 2019) while quantifying the function of stormwater runoff by calculating the predicted storage of water in conventional concrete tanks and its flow through a storm drain is a common practice. Incorporating varying types and sizes of natural landscapes into these calculations presents additional uncertainty. This uncertainty can become a barrier to implementation.

Discounting the potential benefits of green infrastructure due to increased uncertainty stems, in part, from a lack of familiarity with these green systems, and in part, from bounded rationality. Bounded rationality explains that decision-makers are limited in their cognitive resource capacity and time availability to make an optimal decision (Kahneman, 2003). The higher the cognitive load, the greater the potential to deviate from rational assumptions using poorer reasoning, becoming more impatient, and developing greater aversion to perceived risks (Deck and Jahedi, 2015).

A simple and practical approach to reduce cognitive load is through a priming-related event (Jaeggi et al., 2007). Priming is the exposure to a stimulus that has an effect on a subsequent stimulus, without conscious guidance or intention (Molden, 2014). Priming decision-makers to first consider the outcomes of each design option related to the environment and community before considering the monetary cost and risks may help shift how decision-makers prioritize these attributes when evaluating which option to implement. The aim of the study was to explore how priming decision-makers to first think about the benefits of green infrastructure may shift their preferences and if these shifts in preferences change their design decisions. Unique methods to measure cognition were also used. Neuroimaging provides a physiological measure of how priming changes brain function.

The paper begins with more background on the cognitive barriers limiting green infrastructure, dives into the neural mechanisms of priming, and the use of a nascent neuroimaging technique to measure neuro-cognition during design decision making. Measuring neuro-cognition through imaging extends the current understanding of engineering design, offering insight into changes that occur in the brain and how this corresponds to different outcomes in design. The Materials and Methods section outlines the experiment design and data analysis techniques. The changes in neuro-cognition and design decision outcomes are presented in the Results section. The Discussion and Conclusion offer an explanation about the relations between brain and behavior and highlight the added value of measuring engineering neuro-cognition for sustainable design and decision-making.

## Background

### Cognitive Barriers to Green Stormwater Infrastructure

A growing number of cities recognize the multiple benefits that green infrastructure can provide (Zabcik, 2017; Meerow, 2020). Unfortunately, the pace and the scale to implement green infrastructure is not keeping pace with rapidly changing environmental conditions in both developed (Wihlborg et al., 2019; Van Oijstaeijen et al., 2020) and developing countries (Pauleit et al., 2021). This is leaving communities and infrastructure systems more vulnerable when facing challenges associated with climate change (Dhakal and Chevalier, 2017; Hu and Shealy, 2020). Numerous barriers limit the implementation of green infrastructure, such as barriers in policy, governance, resources, and human cognition (Dhakal and Chevalier, 2017; Li et al., 2020). Prior research suggests that cognitive barriers are the most critical to address because most other barriers stem from and are intensified by barriers in human cognition (Dhakal and Chevalier, 2017; Li et al., 2020).

Some of the cognitive barriers that are relevant to stormwater infrastructure include *status quo* bias, risk aversion, and attention bias (Sarah, 2010; Copeland, 2016; Dhakal and Chevalier, 2017; Hu and Shealy, 2020). Engineers describe not wanting to depart from industry norms, to such an extent that they report physical discomfort when non-conforming options are presented to them (Wright, 2011). Colorado’s Urban Water Resources Research Council reported that the leading cause for the lack of implementing green infrastructure is the reluctance among stormwater engineers to try something new (Earles et al., 2009). This pro-conventional mindset and the reliance on the *status quo* persists even when stormwater engineers are presented with new information (Brown, 2014; Li et al., 2020). Changes in their brain are also observable when evaluating green infrastructure options. Neuro-cognitive activation in brain regions generally associated with risk processing were suppressed when decision-makers were evaluating a green infrastructure option compared to a more conventional stormwater infrastructure design option (Hu and Shealy, 2020).

Attention bias further compounds *status quo* bias and risk aversion. Attention bias is a decision-maker’s tendency to fixate on function (e.g., stormwater capture/diversion and cost) while neglecting to consider the possible societal and ecological benefits for each design option (Earles et al., 2009; Li et al., 2019, 2020). More cognitive attention on attributes like cost and risk lead to discounting attributes like the benefits to the community and the environment. Attention bias occurs even when engineers, developers, and end-users consistently rank social and environmental outcomes above economic value as the most critical to project success (Zhang and El-Gohary, 2016). The lack of attention toward the community and the environmental impact during stormwater design seems irrational, even from a neo-classical perspective, when considering stakeholders value these attributes over others. Yet, cost to construct and perceived risk persist as reasons against implementing green stormwater solutions (Green-Nylen and Kiparsky, 2015; Li et al., 2020).

Shifting the focus from cost and risks to the benefits that green infrastructure can provide may help balance the weighting of these attributes during the stormwater design decision-making process. One approach to help encourage more balanced cognitive attention on the benefits of green infrastructure is through priming. Priming is a form of implicit and unconscious memory (Hauptmann and Karni, 2002; She and MacDonald, 2014). Priming-related facilitation processes can influence cognition when people make judgments and decisions (Jaeggi et al., 2007).

### The Use of Priming and Its Neurocognitive Underpinnings

Priming works by using an artifact, exposure, or experience to direct attention and to facilitate the cognitive accessibility of specific content and to motivate a targeted behavior (Komatsu and Ohta, 1985; She and MacDonald, 2014). Research in health (Stockwell, 2009), political science (Lenz, 2009), food science (Ferrari et al., 2019), and environment (Lee et al., 2020a) demonstrate the effectiveness of priming to change human behavior. Engineers have used priming to encourage more consideration and communication of sustainability in the design of mechanical systems (She and MacDonald, 2014).

Studies from neuroscience provide insight into the neuro-cognitive mechanisms that occur through priming (Schacter et al., 2004). The most common finding across multiple priming studies is that it decreases subsequent hemodynamic response in the prefrontal cortex (PFC; Henson, 2003). In other words, the subsequent intended behavior becomes cognitively easier as a result of the priming related event. But other neuroimaging studies also found increased activation in deeper parts of human brain after priming, for example, a recent study found that green logos increased the neural activation in the anterior cingulate cortex (ACC) and consumers who were primed preferred the sustainable products (Lee et al., 2020a). A criticism of prior work on priming in neuroscience is that studies usually use simplistic tasks, such as word-stem completion, masked priming, or semantic priming. There is a gap in understanding how priming may work and influence both the mind and brain of engineers dealing with complex problems during design.

### Neuroimaging to Measure the Effects of Priming

To fill this gap, the research presented in this paper adopted functional near-infrared spectroscopy (fNIRS) to measure the neuro-cognition that occurs during engineering design and decision-making and the changes that occur through a priming-related stimuli. fNIRS is a non-invasive neuroimaging technique that indirectly measures cortical activation through near-infrared light. Light sources emit near-infrared light with different wavelengths into the human cortex. Some light is absorbed by the oxy-hemoglobin (oxy-Hb) and deoxy-hemoglobin (deoxy-Hb) with varying absorption rates in the blood. The light that is not absorbed is reflected and received by the detectors. The change of light is converted into the change of oxy-Hb and deoxy-Hb using a modified Beer-Lambert Law. Oxy-Hb is usually regarded as a proxy for cognitive activation (Herold et al., 2018).

Compared to other neuroimaging techniques (e.g., fMRI or EEG), fNIRS provides relatively good temporal and spatial resolution plus excellent portability in use. Participants can sit or move to complete tasks in a natural environment with an fNIRS cap on their heads. These advantages make fNIRS more applicable in a professional setting to measure the neuro-cognition of engineers during design and decision-making processes (Chrysikou and Gero, 2020). A growing number of studies in engineering research are using fNIRS to measure the neuro-cognition that underpins engineering cognition (Shi et al., 2020; Zhu et al., 2021; Sulbaran and Kisi, 2022). Another benefit of fNIRS is that oxy-Hb is an objective and quantifiable measure for cognitive load. It provides a more direct measure compared to self-evaluation instruments like the NASA-TLX survey questions (Hart, 2006). There is high level of accuracy when classifying the level of cognitive load using the area under the oxy-Hb curve (Gao et al., 2020; Oku and Sato, 2021).

The brain region of interest for design neuro-cognition research is often the PFC. The PFC plays a major role in attention, reasoning, working memory, and decision-making (Asgher et al., 2018). Neural priming literature points to activation change in the PFC related to conceptual processing (i.e., cognitive process relying on learning beyond senses; Gong et al., 2016). The PFC sub-regions, such as dorsolateral PFC (DLPFC), ventrolateral PFC (VLPFC), and medial PFC (mPFC), are integrally involved in the cognitive functions in design and decision-making.

## Research Questions

The research presented in this paper measured both the mind and the brain when civil engineering students who were trained in stormwater design made judgments about a conventional and a green stormwater infrastructure design option. The aim of the study was to explore how priming decision-makers to first think about the benefits of green infrastructure may shift their preferences and if these shifts in preferences are observable in their brain. The specific research questions were:

1. How does priming decision-makers to first consider community and environmental benefits influence their subsequent judgment and decisions?
2. How does this priming intervention change decision-makers’ neuro-cognition when making judgments and decisions?

## Materials and Methods

### Design Scenario and Experiment Design

Forty civil and environmental engineering students (20–28 years old, 16 females, all right-handed) participated in the experiment. All of the participants had previous educational training on stormwater infrastructure design. The students were given a case study adopted from an actual project in Seattle, Washington (Parish, 2017). The case was validated for its content with ten graduate engineering students and it was used and validated in prior study with over 30 engineering graduate students (Hu and Shealy, 2020). During heavy storm events, the excessive stormwater runoff caused flash floods in Venema Creek. This creek was part of a combined sewer overflow system. During heavy rain, sewage would enter the waterway because of the combined sewer overflow. The city proposed two possible options to reduce the stormwater runoff, including (1) a conventional infrastructure design option by constructing storm drains connected to a conveyance pond and (2) a green infrastructure design option by adding a connected bioswale along the roadside and permeable pavement in the residential areas. The engineering students were told to act as infrastructure design consultants and evaluate each option and then make a recommendation.

Half of the participants were randomly selected and assigned to the Intervention Group. Participants in the Intervention Group were primed to think about the sustainable outcomes using the Envision Rating System. Envision provides a comprehensive list of 60 credits under five categories related to sustainability, including Quality of Life, Leadership, Resource Allocation, Natural World, and Climate and Risk (Institute for Sustainable Infrastructure, 2018). To learn about Envision, the Intervention Group read about how the City of Buffalo implemented Envision credits on a previous project. They were then asked to evaluate the sustainability outcomes of the two design options in their case. They were given five of the 60 Envision credits. The five selected credits were “Improve Community Quality of Life,” “Plan for Sustainable Communities,” “Preserve Water Resources,” “Preserve Surface and Groundwater Quality,” and “Improve community resiliency.” These five were chosen because of their connection with stormwater management and this specific case study (Institute for Sustainable Infrastructure, 2018, 2020).

**FIGURE 1:**
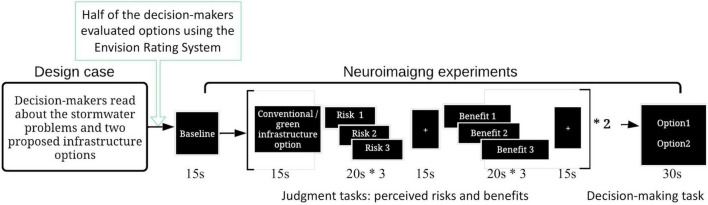
Experiment process. * is the multiplication sign.

Participants were then instructed to make judgments about each of the options relative to their perceived risks and potential benefits. After making these judgments about risks and benefits, they were asked to make a final recommendation for the community. Risks referred to the perceived probability (0–100%) and severity (0–10) in terms of life cycle cost overrun (denoted as risk one in Figure 1), failure in reducing stormwater runoff (risk two), and maintenance schedule not being followed (risk three). These risks were included for evaluation because they were recognized as three critical factors (i.e., how much it costs, how well it reduces runoff, and the level of adoption and maintenance that can be expected) associated with green infrastructure (Montalto et al., 2011).

The benefits referred to the value (0–100) of function in stormwater runoff reduction (denoted as benefit one in Figure 1), community benefits (benefit two), and environmental benefits (benefit three). Each evaluation step in this process lasted for 20 s. The final step asked participants to recommend one of the two infrastructure design options. Participants were given 30 s to make a final recommendation. Prior to the experiment, the design scenario was evaluated for content validity in a pilot study. The pilot study included ten graduate engineering students. The duration for the judgment and decision-making phases were determined through this pilot study. The task prompts were presented to participants using the Pscyhopy software (Pierce, 2018).

**FIGURE 2:**
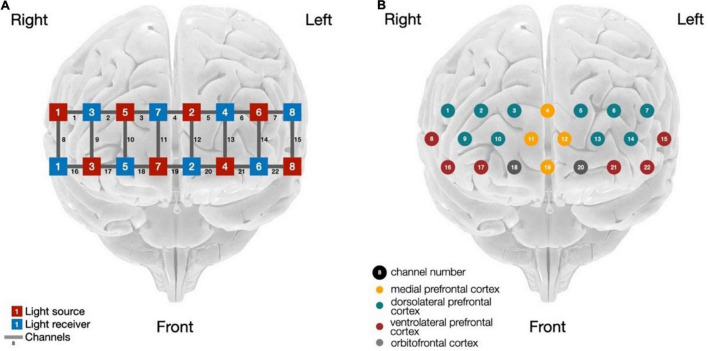
fNIRS sensors and channels: **(A)** Sensors configuration; **(B)** Channels and sub-regions in the PFC.

Neurocognitive activation in the PFC for all participants was measured. The PFC was the region of interest because of its cognitive functions associated with working memory, reasoning, and decision-making (Dias et al., 1996; Asgher et al., 2018). Figure 2 displays the sensor configuration and 22 channels (formed by the combination of a light source and a light detector) that cover several sub-regions in the PFC, including the DLPFC (channels 1, 2, 3, 9, 10 in the right hemisphere, and channels 5, 6, 7, 13, and 14 in the left hemisphere), VLPFC (channels 16 and 17 in the right hemisphere, and channels 21 and 22 in the left hemisphere), orbitofrontal cortex (OFC: channel 18 in the right hemisphere, and channel 20 in the left hemisphere), and mPFC (channels 4, 11, 12, and 19).

### Data Analysis

Both decision outcomes and neurocognitive data were analyzed and compared between the Control Group and Intervention Group. The perceived risks and benefits were scaled and subtracted between the two design options, where positive values represent the perceived risk or benefit of green infrastructure is higher than the conventional infrastructure option. A value of one indicated that they perceived green infrastructure 100 percent over the conventional infrastructure. The perceived benefits and risks recorded from each decision-maker were tested for normality using the Shapiro–Wilk tests. The recorded responses did not meet the normal distribution assumption. So, the Kruskal–Wallis *H* test was used to compare differences in preference between groups. The significant level was 0.05 for the behavioral difference. The effect size was measured using epsilon squared (ε^2^) based on the *H*-statistic. The ε^2^ value below 0.06 was regarded as small. A medium effect size is described as an ε^2^ between 0.06 and 014. Beyond 0.14 was characterized as a large effect size (Tomczak and Tomczak, 2014).

The fNIRS raw data were processed with a bandpass filter (BPF) and Independent Component Analysis (ICA) for noise removal. A low pass of 0.01 Hz and a high pass of 0.1 Hz were used in the BPF (third-order Butterworth filter) to remove instrumental and physiological noise (Naseer and Hong, 2015). A coefficient of spatial uniform of 0.5 was applied in the ICA to remove motion artifacts (Kohno et al., 2007). Only oxy-Hb is reported in the results because oxy-Hb has higher amplitudes and sensitivities to cognitive activities (Hu and Shealy, 2019). Baseline correction was applied to oxy-Hb by subtracting the mean oxy-Hb of the corresponding channel during the resting phase of the experiment.

Based on the blood oxygenation level dependent-local field potential coupling model, positive oxy-Hb corresponds to actively actuated increased blood flow in support of neural activity (Ekstrom, 2010; Bartra et al., 2013). BOLD response (e.g., oxy-Hb) in the PFC implies the allocation of resources and nutrients by the cerebrovascular system (Csipo et al., 2021). The cumulated positive amplitudes of oxy-Hb (i.e., area under the curve) are often used as an indicator of cognitive load or cognitive efforts in the PFC (Manfredini et al., 2009; Agbangla et al., 2017; Suzuki et al., 2018). Similarly, in this study, the positive area under the oxy-Hb curve (AUC) during the task was used as a proxy for cognitive load since it takes both activation level and decision time into consideration when engineering students were making judgments and decisions.

Participants were classified based on their group (i.e., the Control or Intervention Group) and their recommendation choice (i.e., recommending either the green or conventional infrastructure). Normality assumptions were examined using the Shapiro–Wilk test. One-way ANOVAs and *post hoc* Tukey tests were used to compare the cognitive load between the two groups of participants and between participants making different recommendations for the stormwater infrastructure design option. The effect size was measured using partial eta squared (η^2^). A value greater than 0.138 was characterized as a large effect (Tomczak and Tomczak, 2014). The confidence interval was 0.05 but values of less than 0.1 are also noted.

## Results

### Priming Engineers to Consider Sustainability Increased the Perceived Benefits They Believed Green Infrastructure Could Provide

**FIGURE 3:**
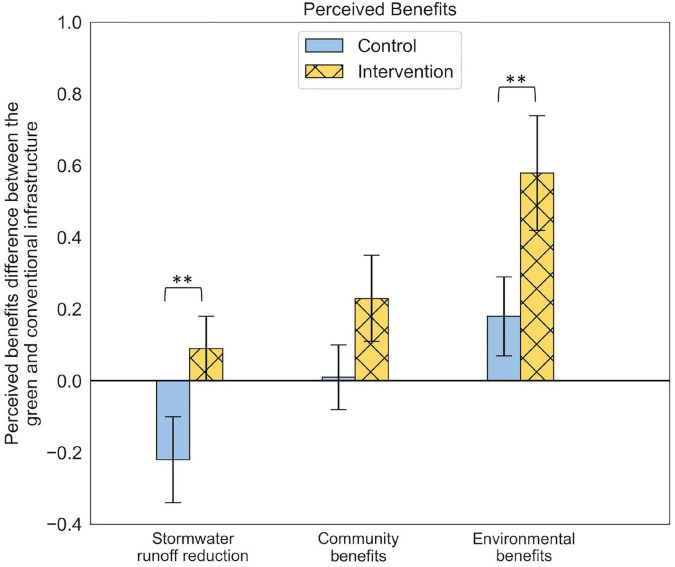
Perceived benefits difference between the green and conventional infrastructure. ^**^p < 0.05.

The Intervention Group perceived the green infrastructure design option as more beneficial in stormwater runoff reduction, providing benefit for the community, and for the environment in comparison to the conventional infrastructure design option (see yellow dashed bars in Figure 3). The Control Group perceived the green infrastructure design option as less beneficial in terms of stormwater runoff reduction compared to the conventional infrastructure design option but slightly more beneficial for the environment (see blue bars in Figure 3). The difference in perceived benefit between the Intervention and Control group was significant (χ^2^ = 4.63, *p* = 0.04), with a medium effect size. The significant difference occurs between the perceived benefits for stormwater runoff reduction (*t* = 2.17, *p* = 0.04) and the environment (*t* = 2.25, *p* = 0.03).

The priming intervention had no significant effect on the perceived risks between the groups of decision-makers. Both the Control and Intervention groups equally perceived green infrastructure as riskier in terms of the potential for life cycle cost overrun, stormwater runoff reduction, and maintenance schedule not being followed. Priming engineers to evaluate the options using the Envision rating system only influenced the perceived benefits that green infrastructure could provide.

### Priming Increased the Number of Engineers Recommending Green Infrastructure

Priming decision-makers to first consider sustainability outcomes prior to making judgments about risks and benefits significantly (*p* < 0.001) increased the frequency that green infrastructure was the recommended design option. Most engineering students (85%) chose the green infrastructure design option in the Intervention Group. The majority (65%) of engineering students in the Control Group recommended the conventional infrastructure option. The effect size between the groups was large (ε^2^ = 0.26). The distribution of recommendations between the cohorts of engineering students is presented in in Table 1.

**TABLE 1:** Distribution of engineering students recommending green or conventional infrastructure.

Number of choices (percentage)	Green infrastructure (percentage)	Conventional infrastructure (percentage)	Total	Kruskal–Wallis χ^2^ (effect size ε^2^)
Control	7 (35%)	13 (65%)	20 (50%)	
Intervention	17 (85%)	3 (15%)	20 (50%)	χ^2^ = 10.2 ***
Total	24 (60%)	16 (40%)	40 (100%)	ε^2^ = 0.26

***p < 0.001.

### Priming Reduced Neuro-Cognitive Activation When Evaluating the Perceived Benefits

The effect of the priming intervention is not only observed in the engineering students’ judgments and their recommendations but also in their neuro-cognition. Two sub-regions in the PFC, the right VLPFC, and the left DLPFC, showed significant differences with a large effect size between the groups of students.

**FIGURE 4:**
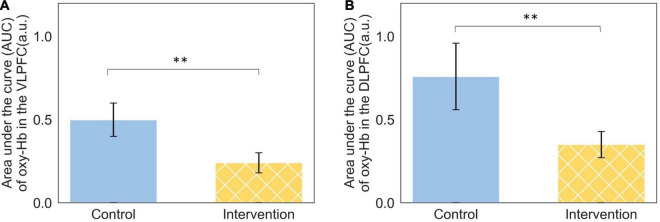
**(A)** Neuro-cognitive activation in the right VLPFC when evaluating the perceived environmental benefits of green infrastructure represented as the area under the curve and **(B)** neuro-cognitive activation in the left DLPFC when evaluating the perceived community benefits of green infrastructure represented as the area under the curve, ^**^p < 0.05.

The students who received the priming intervention and recommended the green infrastructure option demonstrated significantly lower (*t* = −2.32, *p* = 0.03) cognitive activation in their right VLPFC when evaluating the environmental benefits of green infrastructure. In other words, recognizing the environmental benefits of green infrastructure required less cognitive resources after receiving the priming intervention. This is represented in Figure 4A.

A similar reduction in cognitive activation occurs in the left DLPFC when students evaluated perceived community benefits of green infrastructure. The students who received the priming intervention and recommended the green infrastructure option demonstrated significantly lower (*t* = 2.15, *p* = 0.04) cognitive activation in their left DLPFC when evaluating the perceived community benefits of green infrastructure. Recognizing the community benefits of green infrastructure required less cognitive resources after receiving the priming intervention. This is represented in Figure 4B.

### Priming Reduced Neuro-Cognitive Activation When Engineers Made Their Final Recommendation

**FIGURE 5:**
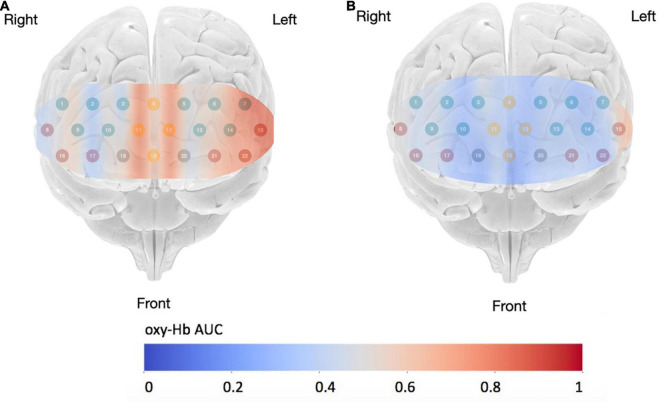
Activation map (AUC) for participants who recommended the green infrastructure: **(A)** Control group; **(B)** Intervention group [Base of brain image copyright© Society for Neuroscience (2017)].

Significant differences in neuro-cognition were also observed when the engineering students made their final recommendation. The group of engineering students who received the priming intervention and selected the green infrastructure option required significantly [(*F*(3,37) = 4.49, *p* = 0.008, and η^2^ = 0.17] less cognitive resources in their PFC to make their selection. In other words, the decision was cognitively easier, requiring less cognitive demand on the decision-maker. Figure 5 includes the brain activation heat maps based on area under the curve (AUC) when the engineering students made their final recommendations.

**FIGURE 6:**
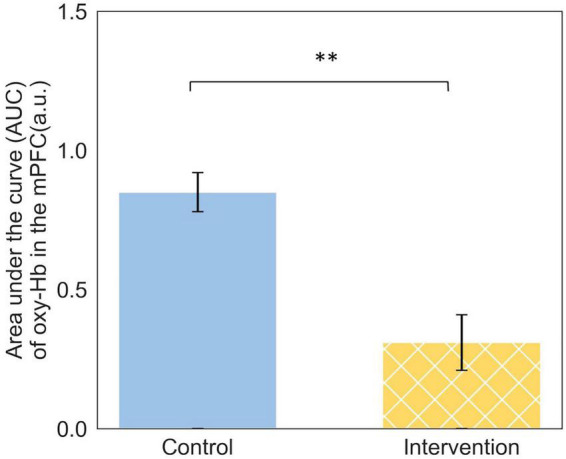
Neuro-cognitive activation in the mPFC when engineering students made their final recommendation for green infrastructure, ^**^p < 0.05.

The significance difference in neuro-cognitive activation when choosing between the conventional and green infrastructure design option occurs in the mPFC. The engineering students in the Control group who recommended the green infrastructure option exhibited significantly (*t* = 2.77, *p* = 0.01) higher levels of cognitive activation in their mPFC region. This difference is illustrated in Figure 6.

## Discussion

This empirical study explored both the mind and brain of engineering students when they made judgments and decisions about stormwater infrastructure. The results demonstrate the effectiveness of priming engineers to think about sustainable design and its effect changing their design preferences. Priming decision-makers to think about the outcomes for the environment and community for each design option using the Envision rating system increased the perceived benefits that green infrastructure could provide over the conventional design option and increased the likelihood that the engineers would recommend the green infrastructure design option.

Two theories help explain why this intervention had an effect on both engineering judgment and decision-making. The first theory is query theory (Johnson et al., 2007). Query theory indicates that the order of query matters. The information presented first tends to be more salient and more heavily weighted in the decision-making process (Weber and Johnson, 2006; Johnson et al., 2007). When decision-makers are asked to first consider the outcomes for each design option for the environment and community, it may increase the cognitive representativeness of these attributes in the decision-maker’s mind and influenced how these attributes are weighted during the judgment and decision-making process.

The second theory is goal priming (Papies, 2016). The priming activity may have unintentionally helped engineers set a goal to achieve greater sustainability on the project. The green infrastructure option scores higher on the Envision rating system. This higher score may have made this option easier to justify in their decision-making. Further research is needed to better understand these underlying mechanisms and their influence on engineering design cognition. These results add to the growing body of literature about how behavioral interventions influence engineering design (Klotz et al., 2018). For example, how modifying the point structure in decision-making tool changes designers’ motivation (Shealy et al., 2019), how prompting engineers to consider life cycle costs increases their willingness to buy more efficient equipment (Delgado et al., 2018), and how industry norms shape engineers’ risk preferences (Hu and Shealy, 2020).

The blending of neuroimaging and choice interventions provides an even deeper explanation for why these types of interventions change engineering cognition. For instance, revealing how and where engineers process risk in their brain and how interventions can change this brain function (Hu and Shealy, 2020). The prior work in Hu and Shealy (2020) tested a behavioral intervention called default effect (McKenzie et al., 2006) by using municipal resolution for green infrastructure to change engineers’ perceived norms for stormwater management. The results from this prior work found the default intervention reduced the perceived risks associated with green infrastructure and this change was also reflected in neural processing for risk in the lateral PFC (Hu and Shealy, 2020). The current work here used the same scenario from the prior research and targeted on changing perceived benefits associated with green infrastructure by using the intervention of priming. The results from this study extend this merging of disciplines presenting new insight into perceived benefit, neural processing for benefits, and the effects of priming.

These interventions may complement each other and future research is needed to understand the combined effect. The prior intervention changed how engineers perceived the risk associated with green infrastructure. Municipal resolutions are formal expressions of opinion by city officials. In this prior study by Hu and Shealy (2020), decision-makers were presented with a resolution signed by city officials stating their support for green infrastructure. This resolution helped reduce perceived risk by signaling the social and industry acceptance for green infrastructure (Hu and Shealy, 2020). The priming intervention may complement this prior intervention because it works differently. Instead of risk reduction, the purpose was to make the benefits of green infrastructure more salient.

The use of neuroimaging provides insight to help understand whether the priming intervention and the municipal resolution influence unique processes in the brain. The prior study by Hu and Shealy (2020) discovered that participants who were informed about the municipal resolution elicited higher brain activation in their right lateral and orbital PFC. The priming intervention led to a reduction in activation in the ventrolateral (VLPFC) and DLPFC. These occurrences of de-activation from the municipal resolution and activation from the priming intervention take place in distinct regions of the brain, yet, are both consistent with the literature (Henson, 2003; Gong et al., 2016; Lee et al., 2020b). When risk decreases, decision-makers with a low risk tolerance elicit higher levels of oxygenated blood in their right lateral PFC (Tobler et al., 2009). Similarly, a reduction in activation in the PFC was previously observed when priming participants in both a learning and reasoning task (Gong et al., 2016; Lee et al., 2020b).

Simply stating de-activation and activation in the PFC does not provide sufficient explanation or context about why these differences may be occurring. Specifically, when evaluating the environmental benefits, the engineering decision-makers who received the priming intervention and selected the green infrastructure option elicited significantly less neuro-cognitive activation in their VLPFC. Previous literature suggests that the VLPFC is generally involved with active memory retrieval processes (Cadoret et al., 2001), when resolving response conflict (Mitchell et al., 2008), and self-referential processing (Northoff et al., 2006; Herwig et al., 2012). A possible explanation is the Intervention Group already recognized many of the benefits from the green infrastructure option as a result of the priming intervention. This recent memory was more accessible, more easily retrieved, and required fewer cognitive resources implied by the reduction in activation in the VLPFC.

Prior literature also mentions that the VLPFC is a critical brain region when processing sustainable decision-making and environmental concerns because of its cognitive function in self-referential processing (Goucher-Lambert, 2017). Self-referential processing refers to the thoughts where one attempts to image another’s thoughts and how others may interpret their own decisions. The engineers in the Control Group who chose the green infrastructure option may have required more cognitive resources in their VLPFC for self-referential processing. Select the green infrastructure option is not common practice and may have required engineers to consider how others may interpret their own judgments. Engineers in the Intervention Group, may have felt more confident about their judgments and recommendation for the green infrastructure option using the Envision Rating System as justification.

A reduction in neuro-cognitive activation was also observed in the DLPFC. Engineers in the Intervention group exhibited less cognitive activation in their DLPFC when making judgments about the community benefits for the green infrastructure design option. The DLPFC is generally is associated with cognitive control (MacDonald et al., 2000) and value encoding (Hutcherson et al., 2012). A prior study found this cognitive control function in the DLPFC modulates social cognitive processing and influences how people process and respond to social information (Lee and Harris, 2013; Hall et al., 2018). The results presented in this study provide supporting evidence for this connection between the DLPFC and social cognitive processing. The reduction in neuro-cognitive activation for participants in the Intervention Group who chose the green infrastructure option might suggest that they already recognized the community benefits and therefore required less elicitation of this region of their PFC to recognize this connection.

Prior studies, the coordinated activation of the DLPFC and the OFC during decision-making has correlated with value encoding (Wallis and Miller, 2003; Lin et al., 2020). Another explanation for the difference in the DLPFC between groups of engineers is that for those who chose the green infrastructure in the Control Group, they needed to subjectively assign a higher value to community benefits that the green infrastructure option provides. This process may have required allocating more neuro-cognitive resources to the DLPFC. The Envision rating system may have helped the Intervention Group assign this value early in the design and decision-making process. Future research should further explore the correlation between the DLPFC, the OFC, and the encoded values related to community benefits.

Change in neuro-cognition was also observed when the engineering decision-makers made their final recommendations for green infrastructure. The mPFC is associated with mediating decision-making in reward-guided learning (Rushworth et al., 2011), making associations (Euston et al., 2012), and reputation in social cognition (Izuma et al., 2010). A possible explanation for the significantly higher cognitive load among the Control Group is that there was a higher demand on neuro-cognition when making associations between the green infrastructure option and the “reward” for selecting this option. One of the associated “reward” of the green infrastructure design is the higher Envision score and this may have been easier to recognize among the Intervention Group and more easily recalled.

The mPFC is also associated with reputation representation in social cognition. So, another possible explanation for the observed difference in neuro-cognition is that decision-makers in the Control Group, who recommended green infrastructure may have felt that their choice deviated from the industry norm and this was expressed with higher neuro-cognitive activation. This is consistent with prior literature (Harris et al., 2016; Hu and Shealy, 2020). For these decision-makers to choose the green infrastructure option, it demanded more neuro-cognitive activation. Reframing the perceived social norms of sustainable design can lead to a significant shift in how engineers’ perceive options, their willingness to implement more sustainable design, and reduction in neuro-cognition (Harris et al., 2016; Hu and Shealy, 2020). Future research should further explore how reframing social norms can have effect on social cognition and how this can shape engineering design (Kinzig et al., 2013).

## Limitations and Future Direction

There were several limitations in this study. The first limitation was that a hypothetical decision-making scenario was used. The recommendations made by the engineering students had no influence on a real-world project. However, the benefit of this experiment was the larger collection of data from multiple decision-makers. Generally, one infrastructure project does not include 40 engineers. This larger sample provided greater statistical power and the ability to measure the effect of priming on both their design cognition and neuro-cognition. Although there was no effect of the engineers’ design decisions on a real-world project, the design decision scenario was modeled on a real-world project in the City of Seattle (Parish, 2017). It provided real-world context and an actual problem that stormwater engineers had to address. The content of the case was also validated in a prior study with over 30 engineering graduate students (Hu and Shealy, 2020).

The period of time that decision-makers were studied was also a limitation. What are the temporal effects of priming? How long are changes in neuro-cognition observable? Even without knowing the answer to these questions, the results presented in this paper can help inform infrastructure planning and design. Early design decisions have an effect on subsequent, future design decisions (Kolltveit and Grønhaug, 2004). Prompting engineers to consider the benefits of their design options for the environment and community changed their mindset, which may lead to future changes and a cascading effect on subsequent design choices. More research on the temporal effects of these interventions is needed but there appears little downside to implementing this type of intervention. This intervention is relatively low-cost compared to the design and construction cost of these physical systems and can be applied quickly in early phase design meetings that occur for stormwater infrastructure.

The sample population is also a limitation. Engineering students might make decisions differently from professional engineers. However, they represent a growing body of novice engineers that will, in the near future, contribute to similar real world design decisions. Professional engineers were also previously shown to be more susceptible to these types of choice modifications than students (Shealy et al., 2019). Knowing that priming engineering students to first consider the benefits of green infrastructure influences their subsequent judgments and design recommendations, allows for future research to replicate the experiment with professionals that likely approach the problem with stronger held biases, more experience with conventional infrastructure, and pre-established preferences for specific design alternatives.

Another limitation was the engineering students were all trained in the United States. While the use of green infrastructure is not common practice in the United States, it is also not an unfamiliar concept. Engineers that participated in the experiment were able to recognize the benefits it could provide once they were prompted through the intervention. These results can provide useful information for other regions with similar accepted norms and practices in engineering. The pace of green infrastructure adoption global is not equal and thus the intervention may not provide similar outcomes when awareness among decision-makers differs (Dhakal and Chevalier, 2017; Li et al., 2020; Pauleit et al., 2021). For example, in countries or regions where green infrastructure encounter fewer cognitive barriers, this type of invention may provide less benefit.

The sample size of 40 provided sufficient statistical power and exceeded the average sample size of prior decision-making studies that implemented fNIRS, which was 28 participants (Hu and Shealy, 2019). The results were significant and a *post hoc* power analysis for one-way ANOVA was performed in G*Power 3 (Faul et al., 2007) to confirm a statistical power of 87%. This is stronger than the conventional acceptable power of 80% (Yarkoni, 2009). This sample was sufficient to measure the effect of the priming intervention. A larger sample could provide the ability to explore additional factors, such as age, gender, or prior experience, that might influence judgments and neurocognition. Computational models such as discrete choice modeling could help to provide more comprehensively analysis about the effects of these factors on design decisions.

Recognizing that design decisions and neuro-cognition are both influenced by this type of intervention helps to inform engineering design, but it is limited in the advancement of neuroimaging. Replicating the study using fMRI can provide an even more complete picture. For instance, additional brain regions like the ACC likely contribute to risk and reward processing (Mulert et al., 2008; Fukunaga et al., 2012). This study was limited to the cortex of the PFC because of the instrumental approach (Hu and Shealy, 2020; Hu et al., 2021). However, using fMRI presents its own unique limitations. Decision-makers must lay inside of an fMRI machine instead being in a professional setting where these types of engineering decisions generally occur. What is the effect of this change of environment on engineering design cognition? This conflict between whole head measurement and an unrealistic environment is a challenge with all neuroimaging studies and why fNIRS seems to currently provide the greatest opportunity for merging engineering design and neuroscience (Ferrari and Quaresima, 2012).

## Conclusion

The increase in perceived benefit for green infrastructure and the reduction in neuro-cognitive activation provides new empirical evidence about the benefits of priming engineers to think about sustainability. Priming engineers to think about sustainable outcomes before evaluating other factor like cost and risk significantly changed their preferences and willingness to recommend the green infrastructure design option. First considering the sustainable outcomes of the design options reduced subsequent neuro-cognitive activation in the engineers’ VLPFC and their DLPFC. The priming intervention also reduced neuro-cognitive demand in the mPFC when making a final recommendation between the green and conventional infrastructure option. These results provide new insights about how modifying the design decision-making process can make the more sustainable design option easier to evaluate and implement. Small changes in how options are presented and the type of information used for evaluation shapes preferences and decisions. These changes are supported with evidence for neuro-cognition. Future research can begin to explore new avenues of research that integrate other behavioral interventions in decision-making process to make more sustainable design options more salient, cognitively easier to recognize, and ultimately increase the frequency these options are recommend for design and construction.

## Data Availability Statement

The raw data supporting the conclusions of this article will be made available by the authors, without undue reservation.

## Ethics Statement

The studies involving human participants were reviewed and approved by Virginia Tech Institutional Review Board (IRB). The participants provided their written informed consent to participate in this study.

## Author Contributions

MH and TS contributed to conception and design of the study. MH performed the data collection and analysis and wrote the first draft of the manuscript. TS wrote sections of the manuscript. Both authors contributed to manuscript revision, read, and approved the submitted version.

## Conflict of Interest

The authors declare that the research was conducted in the absence of any commercial or financial relationships that could be construed as a potential conflict of interest.

## Publisher’s Note

All claims expressed in this article are solely those of the authors and do not necessarily represent those of their affiliated organizations, or those of the publisher, the editors and the reviewers. Any product that may be evaluated in this article, or claim that may be made by its manufacturer, is not guaranteed or endorsed by the publisher.
